# Identification of intratumor bacteria-associated prognostic risk score in adrenocortical carcinoma

**DOI:** 10.1128/spectrum.03727-23

**Published:** 2024-02-29

**Authors:** Linyi Tan, Dengwei Zhang, Yong-xin Li, Yuqing Li, Ting Guo, Yang Sun, Ning Li, Chenchen Feng

**Affiliations:** 1 Department of Urology, Huashan Hospital, Fudan University, Shanghai, China; 2 Department of Chemistry and The Swire Institute of Marine Science, The University of Hong Kong, Hong Kong, China; 3 Department of Gynecology, Obstetrics and Gynecology Hospital of Fudan University, Shanghai, China; 4 Cancer Institute, Xuzhou Medical University, Xuzhou, Jiangsu, China; 5 Department of Urology, Fourth Affiliated Hospital of China Medical University, Shenyang, Liaoning, China; Huazhong University of Science and Technology, Wuhan, China

**Keywords:** adrenocortical carcinoma, intratumor bacteria, prognosis

## Abstract

A landmark study by Poore et al. showed intratumor bacteria (ITBs) playing a critical role in most cancers by reproduction of The Cancer Genome Atlas (TCGA) transcriptome data. A recent study by Salzberg et al. argued that ITBs, being overstated as a methodology by Poore et al., were problematic. We previously reported that ITBs were prognostic in adrenocortical carcinoma (ACC), a highly aggressive rare disease using data by Poore et al., and here, we aimed to answer whether ITBs truly existed and were prognostic in ACC. ACC samples from our institutes underwent 16S rRNA sequencing [adrenocortical carcinoma blocks from Huashan Hospital and China Medical University (HS) cohort]. The ITB profile was compared to TCGA data processed by Poore et al. (TCGA-P) and TCGA data processed by Salzberg et al. (TCGA-S), respectively. The primary outcome was overall survival (OS). A total of 26 ACC cases (HS cohort) and 10 paraffin controls were sequenced. The TCGA cohort encompassed 77 cases. Two and four amid the top 10 abundant genera in HS cohort were not detected in TCGA-P and TCGA-S, respectively. Neither was alpha or beta diversity associated with survival nor could ACC be subtyped by ITB signature in the HS cohort. Notably, a five-genera ITB risk score (*Corynebacterium*, *Mycoplasma*, *Achromobacter*, *Anaerococcus*, and *Streptococcus*) for OS trained in the HS cohort was validated in both TCGA-P and TCGA-S cohorts and was independently prognostic. Whereas ITB signature on the whole may not be associated with ACC subtypes, certain ITB features are associated with prognosis, and a risk score could be generated and validated externally.

**IMPORTANCE:**

In this report, we looked at the role of ITBs in ACC in patients with different race and sequencing platforms. We found a five-genera ITB risk score consistently predicted overall survival in all cohorts. We conclude that certain ITB features are universally pathogenic to ACC.

## INTRODUCTION

Adrenocortical carcinoma (ACC) is a rare disease with occult heralding symptoms. Though curable at the localized stage, most patients are diagnosed at an advanced stage, which confers dismal survival. Identification of novel prognostic markers is urgently needed.

In recent years, next-generation sequencing has brought surprise in cancer studies when scholars identified non-human reads that could be mapped to microbial signature (1). Intratumor microorganisms, especially intratumor bacteria (ITBs), have gained the most attention, given their abundance in comparison to fungi, archaea, or virus. ITB signature has shown a prognostic effect in a variety of cancer types, including gastric cancer (2), colorectal cancer (3), and hepatocellular cancer (4), establishing the microbiome as a novel omics or “second genome” of cancer (5).

Unlike gene-association studies, microbial signature in cancer is largely impacted by contamination, especially in organs conventionally regarded as “sterile” (6). In breast cancer, for instance, only one to two ITBs can be spotted in every ~10,000 cells scanned (7). This makes identification of true ITBs in cancers with low biomass substantially challenging as a vast majority of microbial reads detected are in fact contaminants (8). Nonetheless, since the study of the cancer microbiome is a newly emerging field, a standardized decontamination process is not yet available. Recently, even the landmark study by Poore et al. (1), reproducing RNA-seq data in The Cancer Genome Atlas (TCGA) cohorts to establish an intratumor microbial biomarker, has been challenged recently for potential fallacy in the decontamination process (9).

Our group has been the first to report ITB signature in ACC (10). Using 16S fluorescence *in situ* hybridization (FISH) and lipopolysaccharide (LPS) staining, we observed that the existence of ITBs in ACC and reproduction of TCGA-ACC data set using microbial reads converted by Poore et al. (1) yielded an independent prognostic signature that supersedes the current staging system of ACC (10). Interestingly, a recent report by Cantini et al. showed an association between ITBs and response to mitotane, a adrenolytic medication used in advanced-stage ACC (11).

Together, ITBs could be playing important roles in ACC yet to be elucidated, whereas ITB composition could largely be impacted by contamination, sequencing technique, and even race (12). We thus aim to evaluate whether ITB signature could be extrapolated independently. In the current study, we have sequenced our own ACC samples with a rigorous decontamination process. We owe great thanks to Salzberg et al. (9) for providing their reproduction of TCGA-ACC data. Here, we have developed an ITB risk score in our own cohort and validated our findings in a TCGA data set processed by two top groups (1, 9), where all cohorts differ in sequencing technique, patient race, and processing algorithm.

## MATERIALS AND METHODS

### Sample collection

A total of 26 formalin-fixed paraffin-embedded (FFPE) ACC blocks from Huashan Hospital and China Medical University [termed adrenocortical carcinoma blocks from Huashan Hospital and China Medical University (HS) cohort] were included. Patients underwent surgical removal of tumors between 2014 and 2022. All samples were re-accessed by an independent pathologist for validation of pathological diagnosis.

### 16S rRNA sequencing

All samples were sequenced in one batch. Ten FFPE blocks randomly chosen from the 26 cases were sequenced as control. Total genome DNA from samples was extracted using the cetyltrimethylammonium bromide (CTAB) method. DNA concentration and purity were monitored on 1% agarose gels. According to the concentration, DNA was diluted to 1 μg/μL using sterile water. 16S rRNA genes of a distinct V4 region were amplified using specific primer 16S V4:515 F-806R with the barcode. PCR reactions were carried out with 15 µL of Phusion High-Fidelity PCR Master Mix (New England Biolabs), 0.2 µM of forward and reverse primers, and about 10-ng template DNA. Thermal cycling consisted of initial denaturation at 98°C for 1 min, followed by 30 cycles of denaturation at 98°C for 10 s, annealing at 50°C for 30 s, and elongation at 72°C for 30 s, and finally, 72°C for 5 min. The same volume of IX loading buffer (containing SYB green) was mixed with PCR products, and electrophoresis was performed on 2% agarose gel for detection. PCR products were mixed in equidensity ratios. Then, mixture PCR products were purified with Qiagen Gel Extraction Kit (Qiagen, Germany). Sequencing libraries were generated using TruSeq DNA PCR-Free Sample Preparation Kit (Illumina, USA) following the manufacturer’s recommendations, and index codes were added. The library quality was assessed on the Qubit (v.2.0) fluorometer (Thermo Scientific) and Agilent Bioanalyzer 2100 system. Finally, the library was sequenced on an Illumina NovaSeq platform, and 250-bp paired-end reads were generated. Paired-end reads were assigned to samples based on their unique barcode and were truncated by cutting off the barcode and primer sequence. Paired-end reads were merged using FLASH (v.1.2.7, http://ccb.jhu.edu/software/FLASH/) (13), a very fast and accurate analysis tool which was designed to merge paired-end reads when at least some of the reads overlap the read generated from the opposite end of the same DNA fragment, and the splicing sequences were called raw tags. Quality filtering on the raw tags were performed under specific filtering conditions to obtain the high-quality clean tag according to FASTP (14). The tags were compared with the reference database (Silva database, https://www.arb-silva.de/) using UCHIME algorithm (15) to detect chimera sequences, and then the chimera sequences were removed (16) before effective tags were finally obtained.

### Decontamination

Given the retrospective nature of the current study, contamination was inevitable during and after sample embedment. Processing of FFPE samples before sequencing thus maximally conformed to the recommended protocol to minimize additional contamination (17). Besides regular decontamination process for sterile organs (11, 18), we designated an algorithm workflow to remove blacklist contaminations (19, 20): ASVs (amplicon sequence variant) of contamination were identified based on the “decontam” algorithm, using the “isNotContaminant” function based on the “prevalence model”; ASVs with a relative abundance of more than 0.5% in the control FFPE samples were removed; ASVs that appeared in less than 5% of the tissue sample were also removed to avoid contingency. Normalized read counts of ITBs were subject to further analyses for clinicopathological association.

### Processing of TCGA data

We previously retrieved an NR cluster of TCGA data processed by Poore et al. (TCGA-P cohort), which was ready for analyses (10). Data for the TCGA data processed by Salzberg et al. (TCGA-S) cohort were kindly provided by Professor Steven Salzberg, originally retrieved from the TCGA-ACC data set and processed by a pipeline reported by the team recently (9). In brief, the TCGA-S encompassed microbial counts substantially lower than TCGA-P. Caveat should be taken as our group did not have access to raw transcriptome data of TCGA, so that neither data from TCGA-P nor data from TCGA-S were externally validated by us.

### Genomic analysis and visualization

Somatic variants were acquired by the *GDCquery_Maf* function in the R package “TCGAbiolinks” (21) and were visualized by the R package “maftools” (22). The chi-squared test was used for comparing the frequency of variations between subgroups, and the Wilcoxon test was used for measuring the tumor mutation burden (TMB) and copy number variant (CNV) differences.

### Transcriptomic analysis and visualization

The R package “limma” (23) was used for identifying the differentially expressed genes with a cutoff of 1 for log-transformed fold change. The enrichment analysis of differentially expressed genes was performed using *enrichKEGG* and *gseKEGG* functions in the R package “clusterProfiler” (24). The Quantiseq algorithm was used for evaluating immune cell infiltration by the deconvo_tme function in the R package “IOBR.”

### Statistical analyses

Read counts of ITBs were matched to survival data. All statistics were run using R script. Diversity of ITB and subtype probing was investigated as previously reported (10). Log-rank test was used as the univariate test to identify candidate features, which were subjected to least absolute shrinkage and selection operator (LASSO). Cox regression analysis was conducted using the glmnet package. The multivariate Cox proportional hazards model was employed to investigate independence of ITB risk scores. The *P* value of <0.05 was accepted as statistical significance.

## RESULTS

**Fig 1:**
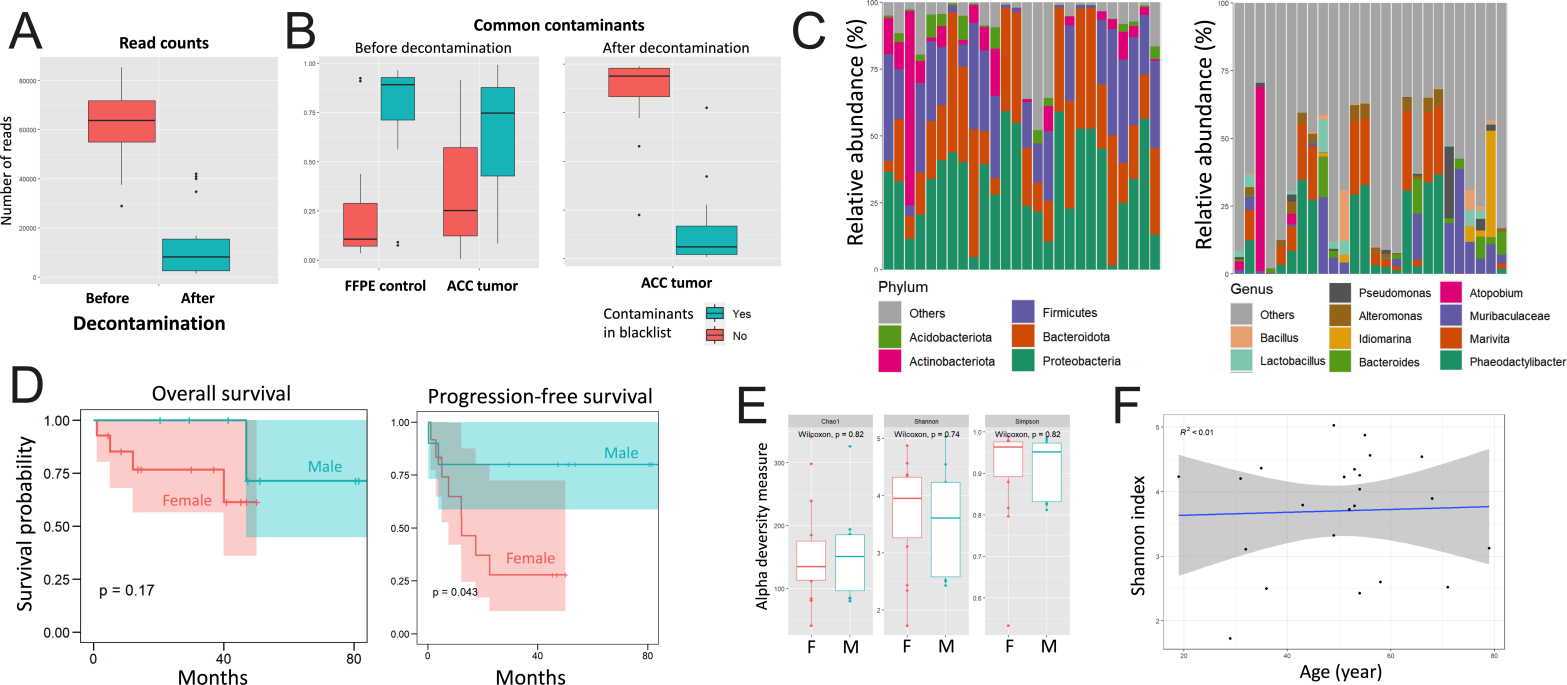
Intratumor bacteria (ITB) exist in adrenocortical carcinoma (ACC). Sequenced by 16S rRNA are (**A**) the drop of read counts before and after the decontamination process; (**B**) the drop of blacklist contaminant after decontamination controlled by FFPE samples; and the composition of top abundant ITBs at (**C**) phylum (top 5) and genus (top 10) levels of 26 cases in the HS cohort. In the HS cohort, shown are the (**D**) overall survival and the progression-free survival, (**E**) three indices indicative of alpha diversity between gender; and (**F**) correlation between patient age and Shannon index. F, female; M, male.

All cases in he HS cohort were of Chinese Han ethnicity. Loss of follow-up occurred in two cases for overall survival (OS) and in four cases for progression-free survival (PFS), respectively. The TCGA cohort encompassed 77 and 72 cases with OS and PFS, respectively. There were 14 female and 12 male ACC patients in the HS cohort at an average age of 51.7 ± 14.3 years. Demographic data for cases in the TCGA-ACC cohort can be found in our previous report (10). Among the five clusters of TCGA-P, we decided to use NR clustering in the current study for validation as it yielded best area under the curve (AUC) in prognosis prediction in our previous model (10). Contamination is the key factor biasing interpretation of ITBs in cancers with low biomass. Using an in-house developed rigorous decontamination process that controlled both for FFPE and blacklist contaminants, we showed a drastic drop in read counts (Fig. 1A). Blacklist contaminants that were highly abundant in both FFPE control and in tumor samples substantially dropped after our decontamination process (Fig. 1B).

Bacterial reads that survived decontamination were presented in relative abundance and annotated phylum and genus levels. Top five abundant phyla are presented in Fig. 1C, four of which, namely, *Actinobacteriota*, *Firmicutes*, *Bacteroidota*, and *Proteobacteria* were present in all cases (Fig. 1C). Taxa varied at genus level between cases (Fig. 1C). Such diversity was also present across cohorts as solely *Bacillus* and *Pseudomonas* were among the top abundant genera in the TCGA-ACC cohort (10). Interestingly, *Pseudomonas* was also present in the study by Cantini et al. (11).

**Fig 2:**
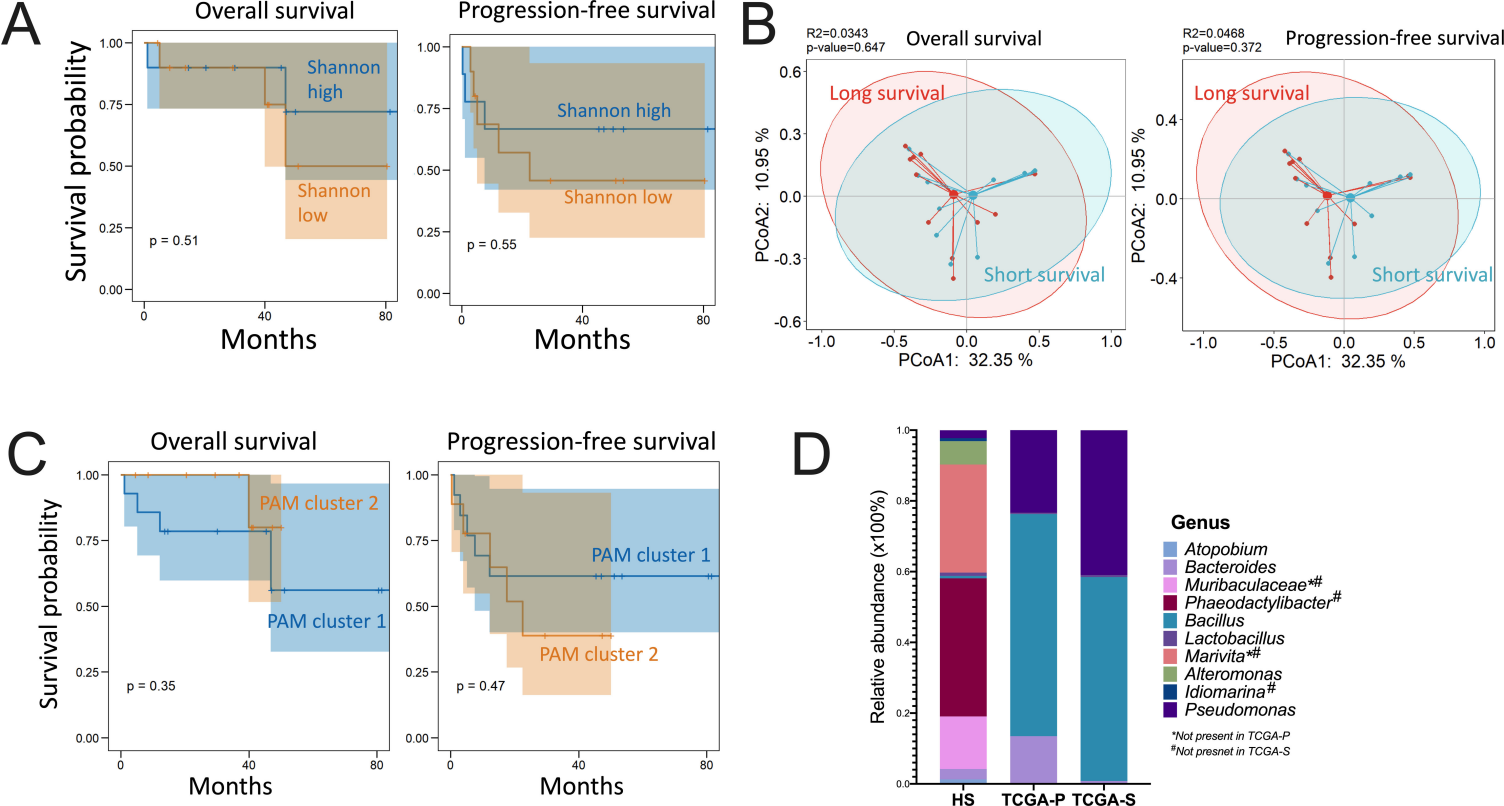
Adrenocortical carcinoma (ACC) cannot be subtyped by intratumor bacteria (ITB) signature. Shown are (**A**) the association between Shannon index and overall survival/progression-ree survival; (**B**) the association between dichotomized survival time by median survival time and beta diversity, and (**C**) the subtyping of ACC using unsupervised partition around medoids (PAM) clustering of all ITB in the HS cohort; (**D**) demonstration of relative abundance of top 10 abundant ITB in the HS cohort composition in two other TGCA cohorts with ITB classified as “other” removed.

As no paired healthy adrenal tissue was available, we compared diversity between demographic parameters. Female patients showed a significantly worsened PFS and a numerically worsened OS in the HS cohort (Fig. 1D). We compared alpha diversity by three indices, but none was altered between sexes (Fig. 1E), neither was Shannon index correlated with age (Fig. 1F). Alpha diversity was correlated with neither OS nor PFS (Fig. 2A). We next explored whether the ITB subtype existed in ACC sequenced with 16S rRNA, though we successfully established a dichotomized signature by reproduction of the transcriptome data of TCGA based on overall survival (10). By dichotomizing according to median length of survival, we did not find differential beta diversity (Fig. 2B). Using ITB signature as a whole also failed to subtype ACC patients according to OS or PFS (Fig. 2C). Surprisingly, we showed here that ITBs, on the whole, were not associated with key clinicopathological parameters of ACC, despite the limited sample size and the rigorous decontamination process.

**Fig 3:**
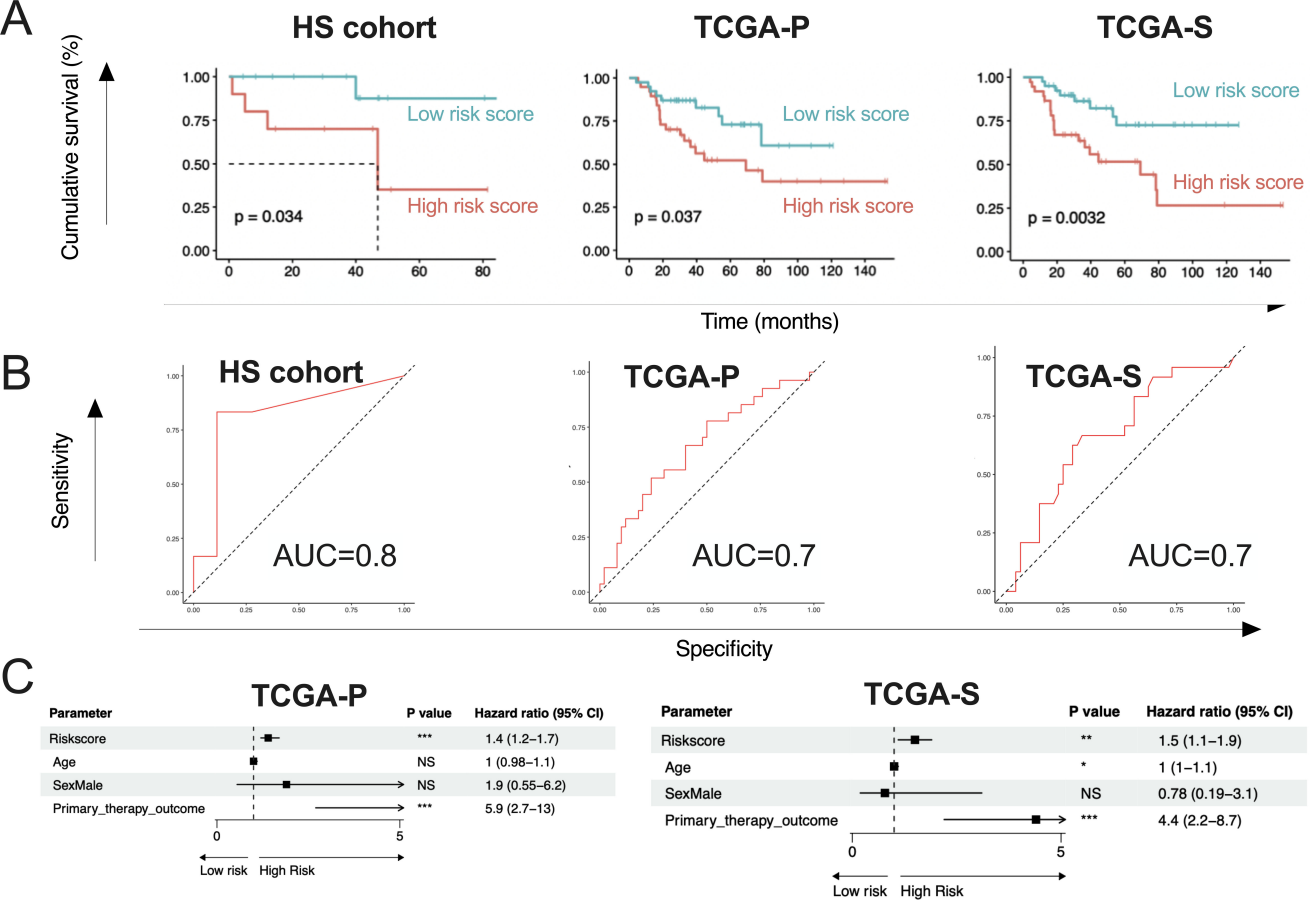
Risk score based on intratumor bacteria (ITB) features is independently prognostic. Constructed using 5 ITB features in Fig. S2, the LASSO model shows (A) Kaplan-Meier curves of ACC cases with high and low risk score compared by log-rank test, trained in HS cohort and validated in TCGA-P/TCGA-S cohorts and (**B**) corresponding receiver operating characteristic curve (ROC) and area under the curve (AUC) profiles; (**C**) forest plot showing independence of the risk score in TCGA-P/TCGA-S cohorts. *P < 0.05, **P < 0.01, ***P < 0.001. NS, not significant.

The discrepancies with our previous reports that ITB signature was not adequately informative to subtype ACC prompted us to answer another question, namely, whether certain ITB features were associated with prognosis in ACC ubiquitously. We first queried the top 10 abundant ITBs in the HS cohort in TCGA-P and TCGA-S, respectively. Of note, two genera (Muribaculaceae and *Marivita*) were not present in the TCGA-P cohort, and two other genera (*Phaeodactylibacter* and *Idiomarina*) were additionally absent in the TCGA-S cohort (Fig. 2D). In general, composition was much similar between the TCGA-P and TCGA-S cohorts than between the HS and TCGA cohorts (Fig. 2D). Standardized Schoenfeld residual validated a LASSO model for feature selection (Fig. S1), which showed five genera (*Corynebacterium*, *Mycoplasma*, *Achromobacter*, *Anaerococcus*, and *Streptococcus*) as risk factors in all three cohorts consistently (Fig. S2). A risk score generated from the five features showed significant prognostication in all three cohorts (Fig. 3A). The score generated an AUC of 0.8 in the HS cohort and 0.7 in both the TCGA-P and TCGA-S cohorts (Fig. 3B). Whereas multivariate analyses showed marginal independence of ITB risk score in HS cohort (*P* = 0.069), it showed independence in both TCGA-P and TCGA-S cohorts (Fig. 3C). We next explored ITB features associated with PFS and identified two genera showing *Paenibacillus* being protective and *Luteibacter* being a risk factor in all three cohorts (Fig. S3). However, we did not obtain a reproducible risk score for PFS with the two features. The best model was trained in the TCGA-P cohort, showing a significant prognostication with a marginal significance in the HS cohort and a numeric difference in the TCGA-S cohort (Fig. S4).

**Fig 4:**
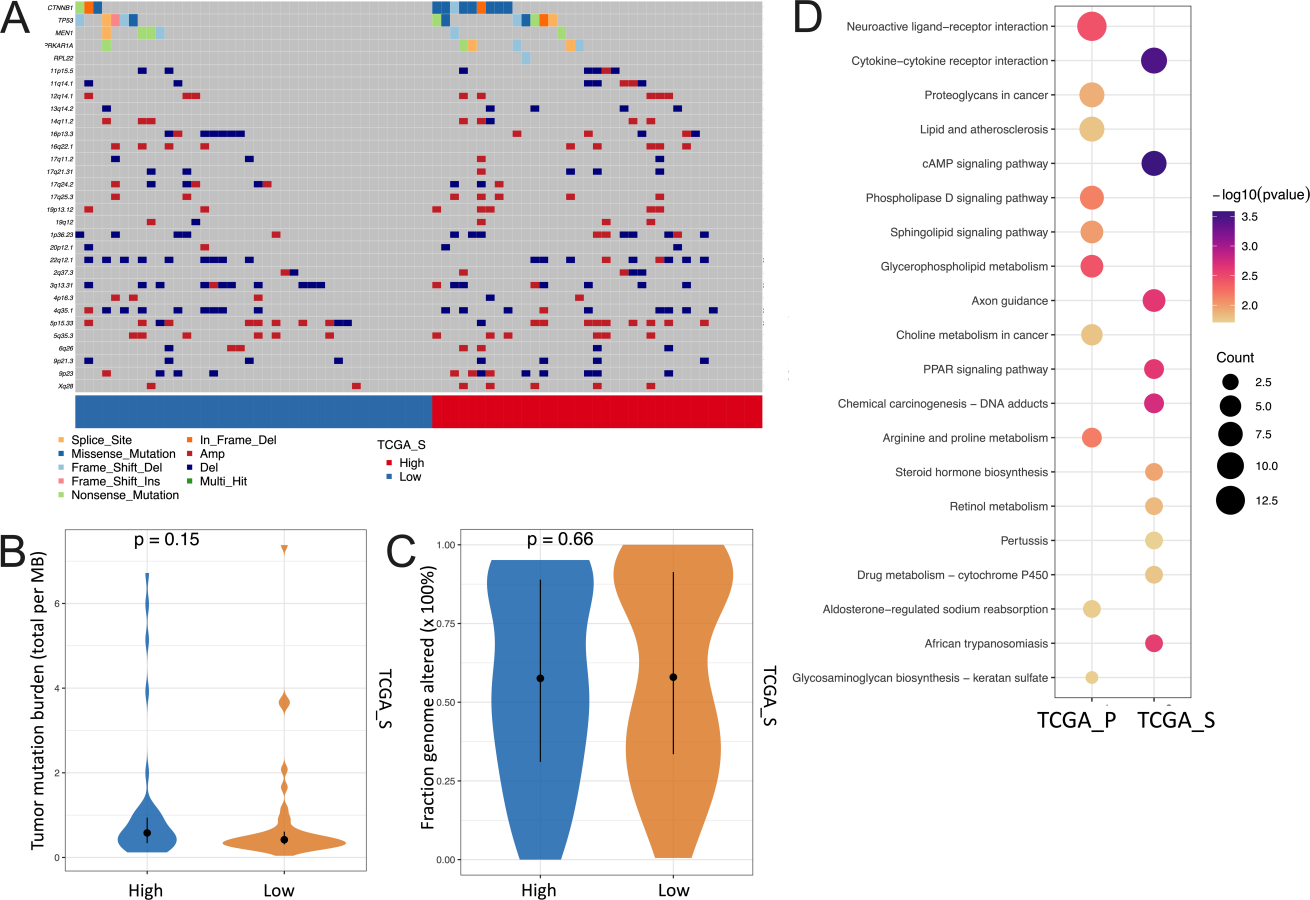
Genomic and genetic alterations in ACC grouped by risk score of OS. (**A**) Waterfall plot showing the distribution of driver genomic events including gene mutation and copy number variation in the subgroup categorized by overall survival risk score in the TCGA-S cohort; violin plot showing the difference of (**B**) TMB and (**C**) fraction genome altered between high- and low-risk score groups in the TCGA-S cohort; (**D**) Bubble plot showing the top 10 pathways enriched by differentially expressed genes between high- and low-risk score groups using the Kyoto Encyclopedia of Genes and Genomes database in the TCGA-P and TCGA-S cohorts. PPAR, peroxisome proliferator-activated receptor; cAMP, cyclic AMP.

Furthermore, due to the better performance of the five-genera risk score in predicting the overall survival, we took advantage of the genomics and transcriptomics data of TCGA to characterize the potential correlation between signaling pathways and microbial community. However, the landscape of the genomic alteration showed rarely discrepant genomic events between the high-risk-score group and the low one for both the TCGA-P (Fig. S5A) and TCGA-S (Fig. 4A) cohorts. Moreover, there was no difference in the TMBs (Fig. 4B; Fig. S5B) and CNVs (Fig. 4C; Fig. S5C) between the two subgroups.

**Fig 5:**
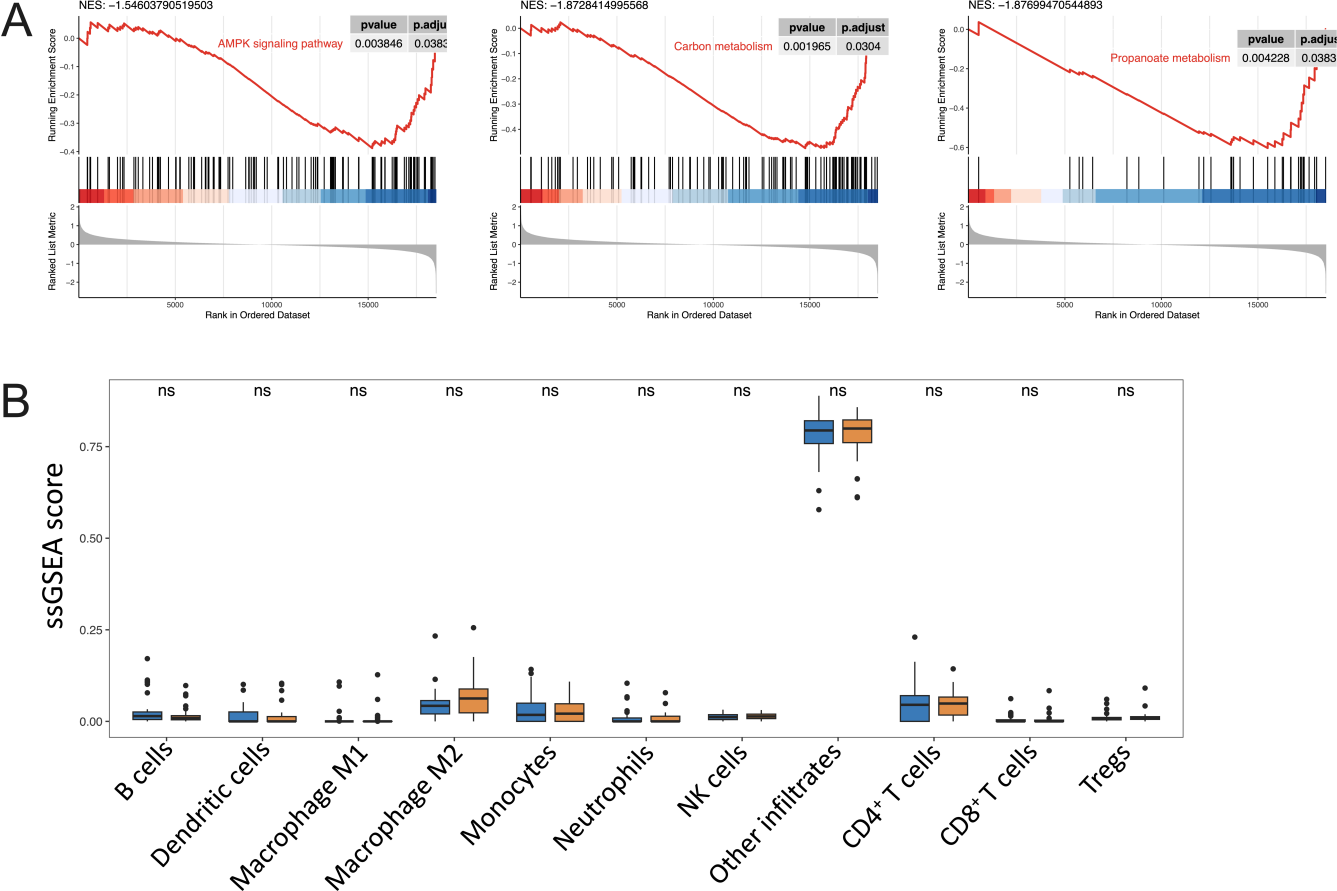
Survival-associated ITB is metabolic in ACC in the TCGA-S cohort. (**A**) The metabolic pathways were differentially enriched between high- and low-risk score groups by gene set enrichment analysis. (**B**) Boxplot showing the difference in the immune cell infiltration scores between high- and low-risk score groups. ssGSEA, single-sample gene set enrichment analysis.

We then explored the differentially expressed genes between the subgroups and found that these genes tend to function in pathways related to metabolism, such as glycerophospholipid metabolism, choline metabolism in cancer, arginine and proline metabolism, and retinol metabolism (Fig. 4D). Also, the gene set enrichment analysis showed that the AMPK signaling pathway, propanoate metabolism, and carbon metabolism were significantly discriminated between subgroups determined by the five-genera risk score, suggesting the metabolic orchestration performed by some intratumor microbiomes (Fig. 5A; Fig. S6A). Of note, we did not observe the difference in the infiltration of the immune cells between the subgroups, implying the tendency of metabolic participation rather than the immune response of the five-genera community in the tumor microenvironment (Fig. 5B; Fig. S6B).

## DISCUSSION

We reported here a five-genera ITB risk score in our own cohort and validated our findings in the TCGA data set processed by the two top groups (1, 9), where all cohorts differ in sequencing technique, patient race, and processing algorithm. Recent debate over the existence and effect of intratumor microbes has drawn much attention. Besides the TCGA microbiome project, another landmark study showing intratumor fungi playing a pathogenic role in pancreatic cancer (25) was challenged that such mycobiome was solely a bystander (26). To sum up, key questions regarding ITBs in cancer include following: (i) is there such a diversity of ITBs in low-biomass cancers? If so, (ii) can the diversity by used as biomarkers (diagnostic, prognostic, and/or response)? If so, (III) do certain ITBs play a causal role in cancer? Our previous study on ACC showed ITB composition could not only differentiate it from other cancers but also subtype the disease according to survival (10). Though such notion was counter-intuitive for the adrenal gland, which is commonly accepted as a sterile organ, though virus was detected therein long ago (27). In order to present such clinical association, microbial data should be quantitatively abundant to generate statistical significance. Whereas microbial decontamination was accepted widely, Salzberg et al. (9) argued contamination of host genetic misclassified as microbial reads could be a problem resulting from high abundance of ITBs in the study by Poore et al. (1) and has provided stringently decontaminated data. Our findings, validated in both cohorts, support the notion that certain ITB features, namely, *Corynebacterium*, *Mycoplasma*, *Achromobacter*, *Anaerococcus*, and *Streptococcus*, rather than microbial diversity on the whole, may play pathogenic roles in ACC. However, features associated with PFS should be intepreted with caveat. Limited sample size in our cohort, lack of consistent statistical significance, and differences in definition of progression all render our investigation of PFS solely exploratory and our results heterogenous.

*Corynebacterium* has been recognized as an important pathogen in immunosuppressive patients, and many non-diphtheria corynebacterial can also be virulent in hospitalized patients. Of note, *Corynebacterium* has become a common infected pathogen in cancer patients in Brazil due to indwelling instruments like catheters and intravenous tubes (28). This corresponds to the notion that most ITBs migrate to tumors via the bloodstream. Mycoplasma on the other hand, has been reported to play a critical role in various malignant tumors (29–31). In fact, the whole ITB research community was mostly, if not entirely inspired by the interesting report on mycoplasma modulating chemoresistance in solid tumor (32). Similar to *Corynebacterium* infection, patients with cancer are also at risk of *Achromobacter* infection due to immunosuppression and the use of prophylaxis with fluoroquinolones (33). *Anaerococcus* and *Streptococcus* were both often reported in cancer microbiome studies, particularly in bladder cancer (34).

However, presence and association do not mean causation (35). Though a robust association between those ITBs and prognosis is shown here in ACC, the causal relation between ITBs and adrenal tumorigenesis remain unknown. Whether ITBs play a commensal or driving role alongside tumor progression depends on human microbiota-associated murine models and microbe-phenotype triangulation (35). Fortunately, a transgenic murine model for ACC has just been reported this year (36), and culturomics is therefore applicable in the future. Alternatively, one may apply patient-derived ITBs for further functional analyses. However, the rarity of the disease may substantially hinder the process, and multicenter collaboration is needed. Once again limited by the rare nature of the disease, we strived to collect ACC cases with relatively complete clinicopathological parameters. The long time span for sample collection rendered many of the patients lost to follow-ups, and thus, only 26 cases were eligible to the current study. Though we managed to follow up more ACC patients that dated further back, their FFPE blocks decayed substantially and were not applicable for 16S sequencing. Another inherent limitation is that ACC samples are extremely large and invasive that no “adjacent” normal adrenal tissue was available. This is a universal problem as the TCGA data also harbor no paired normal tissue. In our previous analysis, we compared data from ACC with those of paraganglioma/pheochromocytoma, given the consideration of similar organ origin and surgical procedure. Though Cantini et al. showed deferential ITBs between unpaired ACC and healthy adrenal tissue (11), we did not adopt such approach as intratissue microbiome vary drastically and interpersonal diversity could even be magnified. Also, we did not measure ITB loads in ACC, as our primary goal was to identify common ITB features associated with prognosis, and loading information could not be profiled in TGGA cohorts. However, as recent studies point out that absolute, rather than relative, abundance plays a more important role in microbiome study (37) and load is a prognostic in nasopharyngeal cancer (38), we are now setting up a new line to evaluate the association between ITB loads and prognosis.

Using the five genera associated with OS, we were able to perform a functional analysis of those features. We found that, unlike in renal cancer in our another project (39), survival-associated ITBs in ACC were not related to immunity but to metabolism. This could result in a different bacterial community between the diseases. Amid the three metabolic pathways, propanoate metabolism is of interest. Propionate is observed to be among the most common short-chain fatty acids produced in the large intestine of humans by gut microbiota in response to indigestible carbohydrates (dietary fiber) in the diet (40, 41). Though it is known to suppress bacterial immune response, its metabolism activation in the presence of those OS-related genera highly suggested that propanoate is catalyzed or metabolized by the bacteria. Certain species of *Corynebacterium* (42), *Mycoplasma* (43), *Achromobactin* (44), *Anaerococcus* (45), and *Streptococcus* (46) were reported to either metabolize or generate propanoate. How propanoate metabolism of the genera impacts on ACC warrants further functional study.

Lastly, we did not put much effort in imaging ITBs in the current study. 16S FISH staining and bacterial LPS staining of ACC samples were shown in our previous report (10) using samples that overlap with the HS cohort. For low-biomass cancer, we tend to consider both LPS and FISH staining could harbor magnified signals from extra-tumor bacterial contamination, a notion supported by a recent report (47). Our findings, together with validation in the TCGA-P/TCGA-S cohorts, has undoubtfully proven the existence and prognostication of ITBs in ACC.

### Conclusion

Whereas ITB signature on the whole may not be associated with ACC subtypes, certain ITB features are associated with prognosis, and a risk score could be generated and validated externally.

## Data Availability

Read counts of un-decontaminated intratumor bacteria were deposited at China National Center for Bioinformation (CRA015044). Codes for reproduction of The Cancer Genome Atlas (TCGA) data processed by Poore et al. (NR cluster) are linked to the GitHub release (https://github.com/ZhangDengwei/ACC_Project). Request for TCGA-adrenocortical carcinoma data processed by Salzberg et al. should be addressed to Professor Steven Salzberg (steven.salzberg@gmail.com). Clinicopathological data may be provided upon request to the corresponding author (C.F.).

## SUPPLEMENTAL MATERIAL

Supplemental figures spectrum.03727-23-s0001.docx Figures S1 to S6.

ASM does not own the copyrights to Supplemental Material that may be linked to, or accessed through, an article. The authors have granted ASM a non-exclusive, world-wide license to publish the Supplemental Material files. Please contact the corresponding author directly for reuse.
