# Supplementary material for: Identification of intratumor bacteria-associated prognostic risk score in adrenocortical carcinoma
Source: Microbiol Spectr. 2024 Feb 29;12(4):e03727-23. doi: 10.1128/spectrum.03727-23 (PMC10986527; doi:10.1128/spectrum.03727-23)
Supplement: Supplemental figures — Figures S1 to S6. [file spectrum.03727-23-s0001.docx]

**Identification of intratumor bacteria-associated prognostic risk score in adrenocortical carcinoma**

Linyi Tan^1#^, Dengwei Zhang^2#^, Yong-xin Li^2^, Yuqing Li^1^, Ting Guo^3^, Yang Sun^4^, Ning Li^5^* and Chenchen Feng^1^*

**Supplementary figures**

**Fig S1.** Dot plot showing the standardized Schoenfeld residual relative to time for each covariate which was used for constructing LASSO COX model, the significance was tested by Schoenfeld test.

**
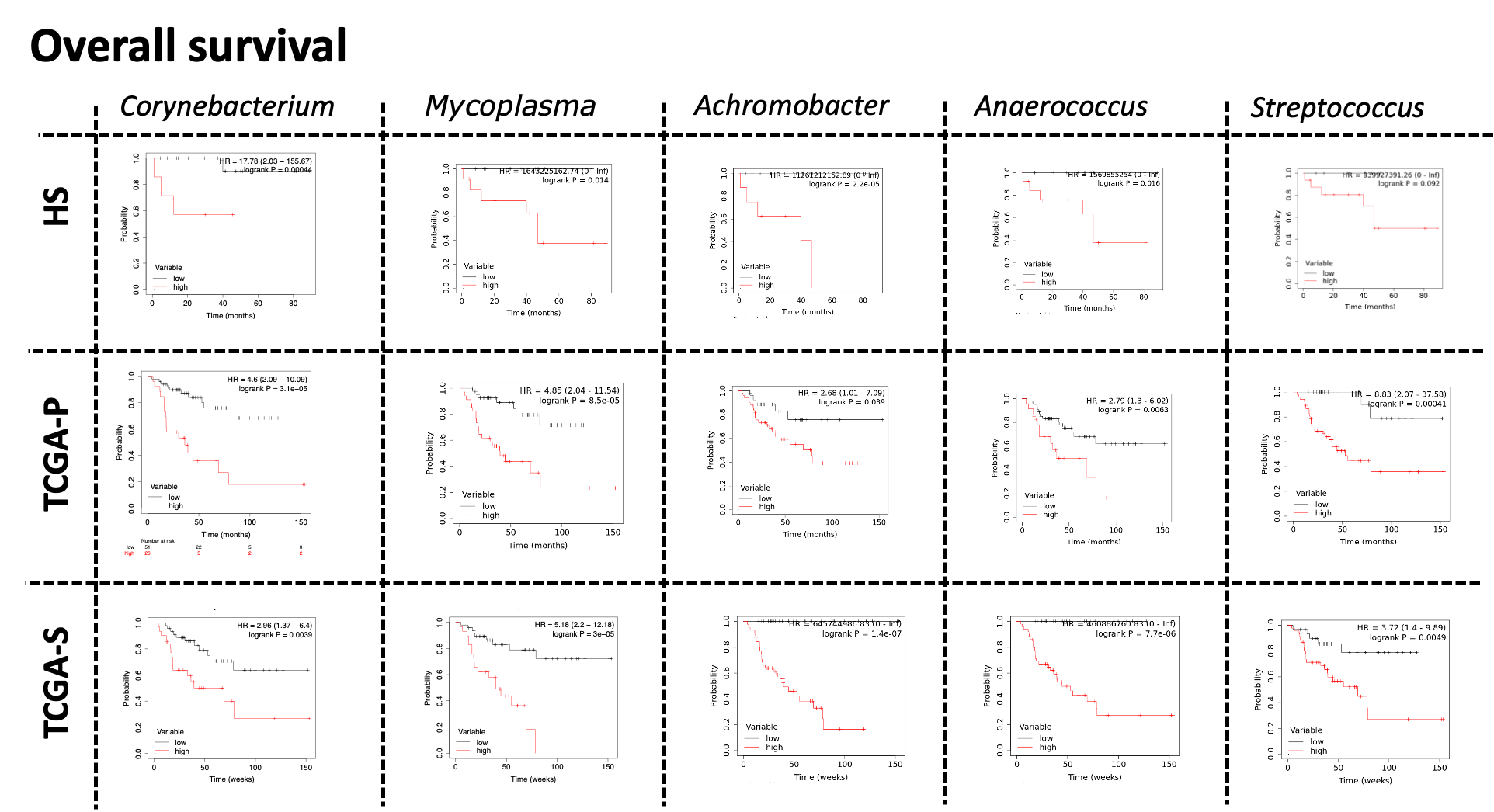
**

**Fig S2.** Intratumor bacterial (ITB) features at genus level showing significant prognostic effect tested by univariate Log-rank. Shown were 5 features all being risk factors for overall survival in HS cohort, TCGA-P cohort and TCGA-S cohort.

**
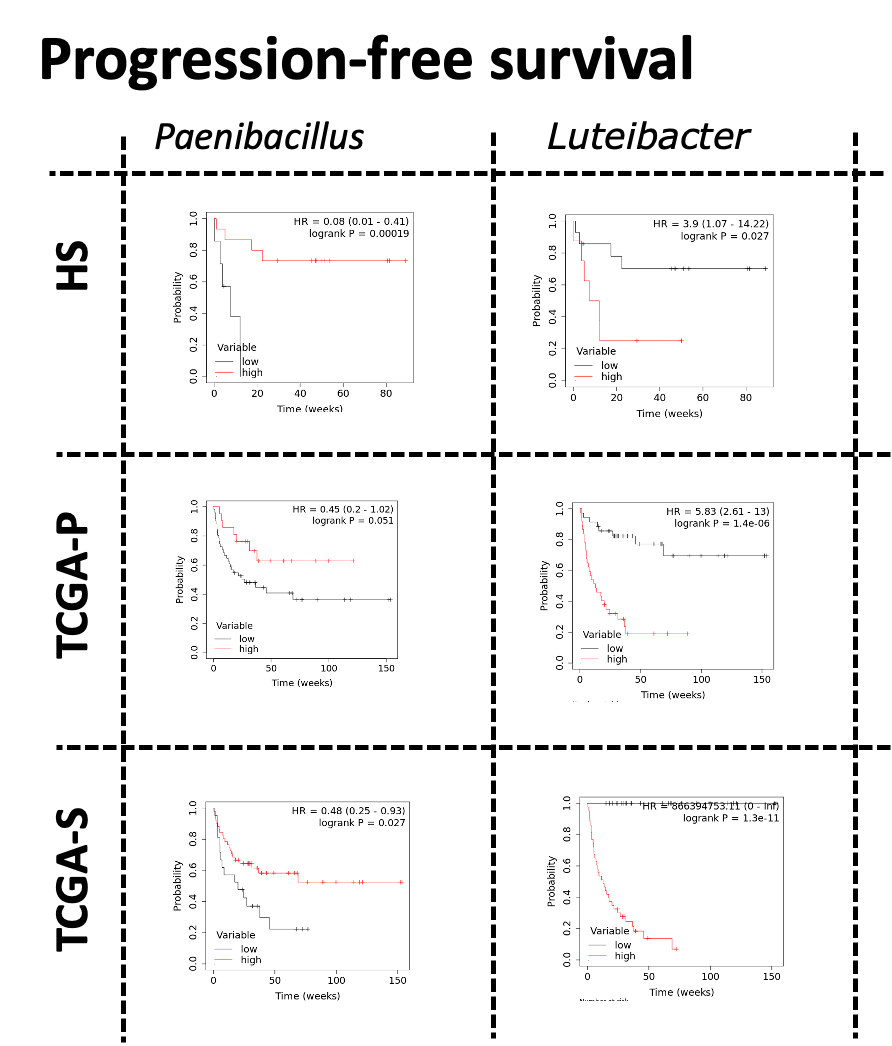
**

**Fig S3.** Intratumor bacterial (ITB) features at genus level showing significant prognostic effect tested by univariate Log-rank. Shown were 2 features for progression-free survival of which one being risk factor and the other being risk factor in HS cohort, TCGA-P cohort and TCGA-S cohort.

**
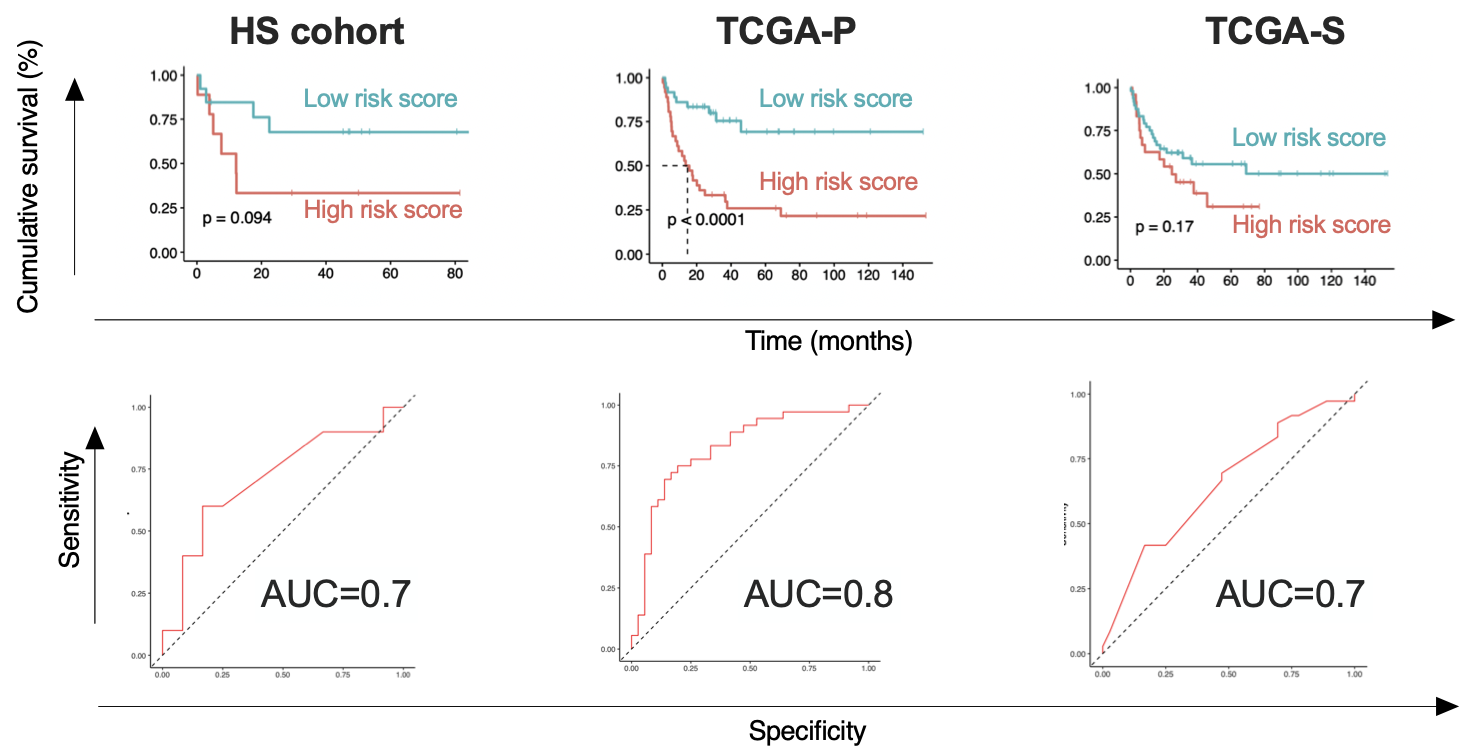
**

**Fig S4.** Intratumor bacterial (ITB) risk scores for progression-free survival (PFS). Shown were 2-genera risk score (*Paenibacillus* and *Luteibacter*) used in LASSO model for PFS prediction, trained in TCGA-P cohort and validated in HS and TCGA-S cohort, with AUCs shown below.


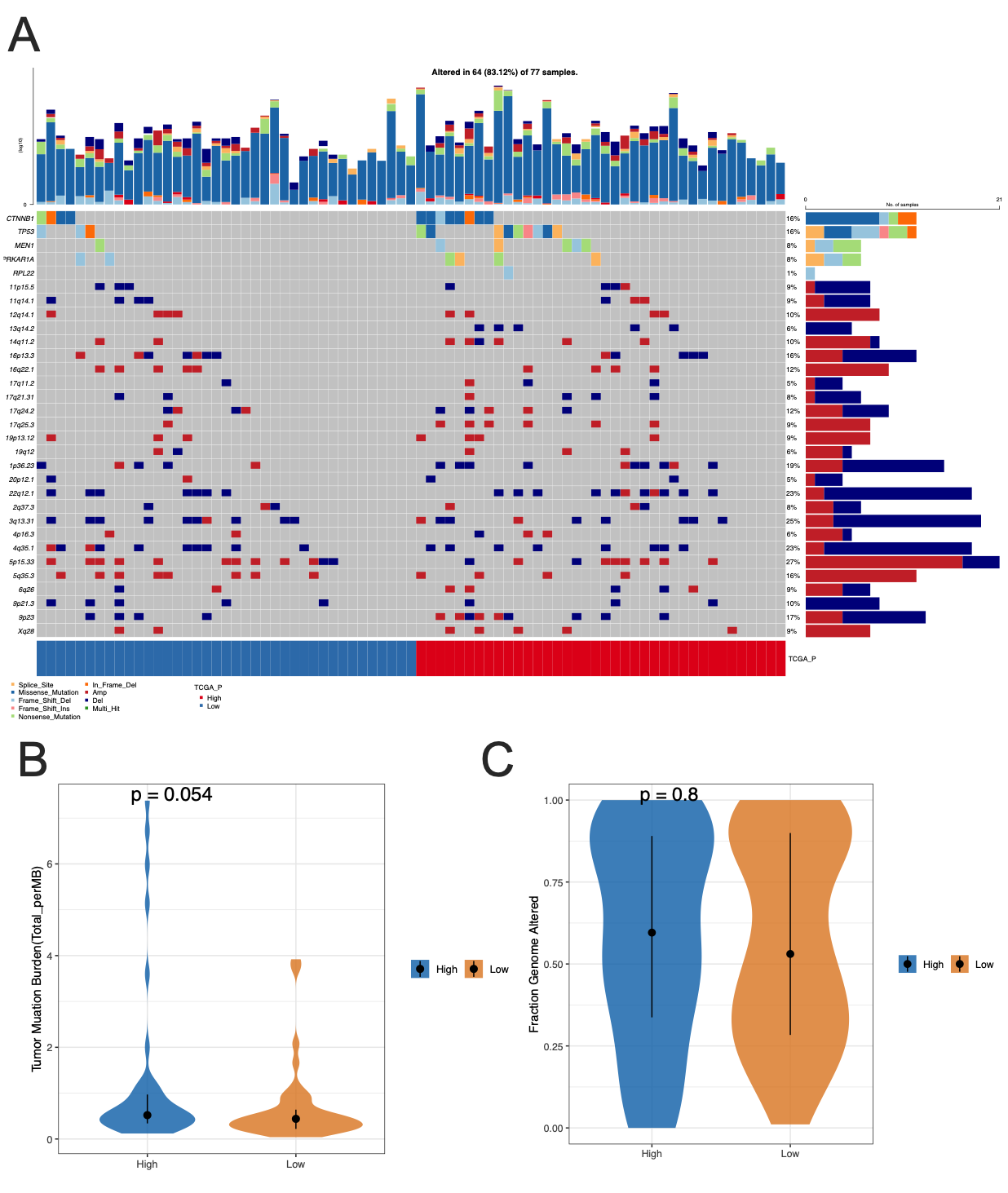


**Fig S5.** Genomic and genetic alterations in ACC grouped by risk score of OS in TCGA-P cohort. (A) Waterfall plot showing the distribution of driver genomic events including gene mutation and copy number variation in sub-group categorized by overall survival risk score; (B) Violin plot showing the difference of the tumor mutation burden (TMB) and fraction genome altered (FGA) between high- and low-risk score group.

**
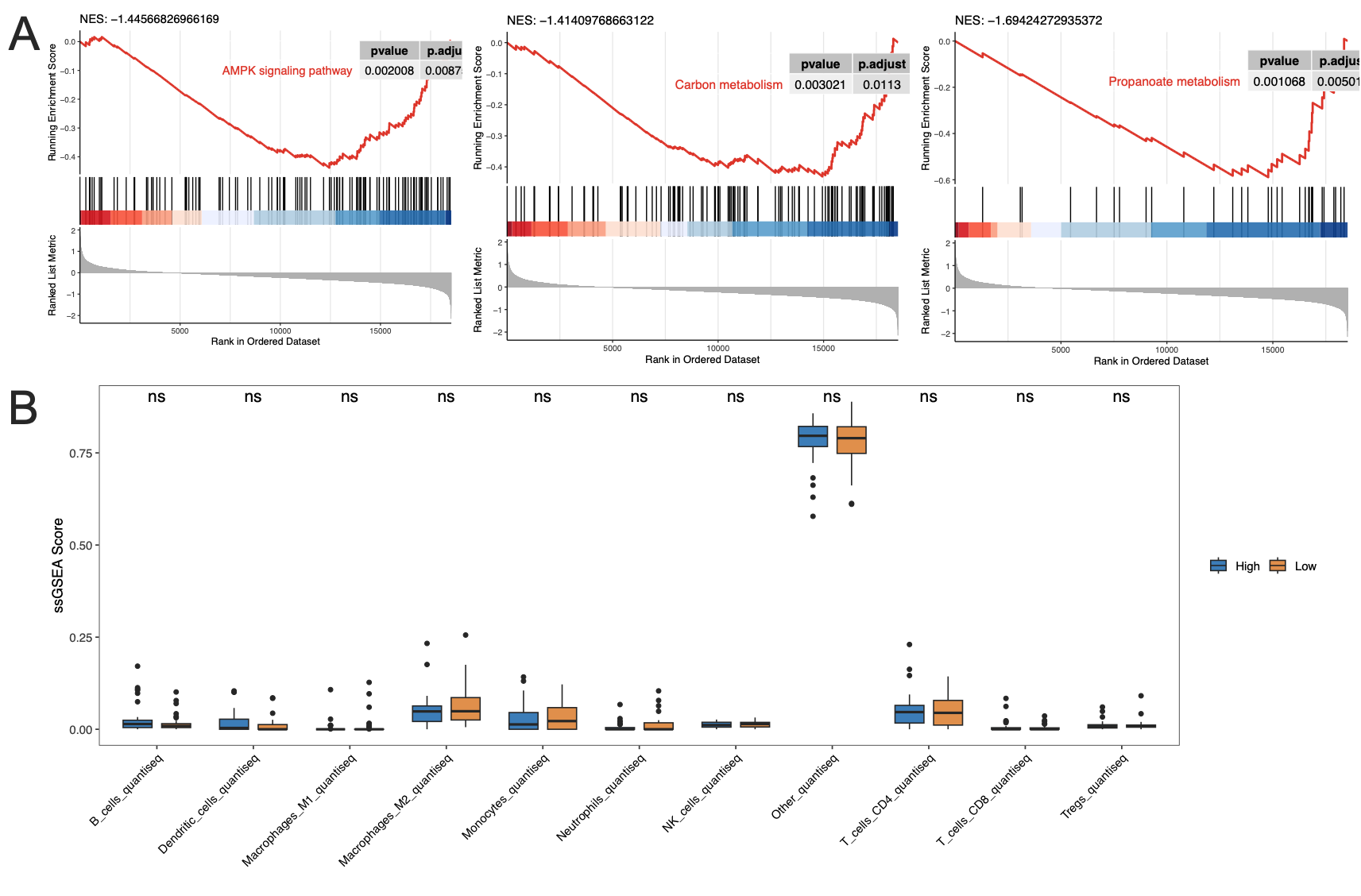
**

**Fig S6.** Functional output in the ITB risk score-grouped ACC cases in TCGA-P cohort. (A) The metabolic pathways differentially enriched between high- and low-risk score group by gene set enrichment analysis. (B) Boxplot showing the difference of the immune cell infiltration score between high- and low-risk score groups.
